# Medicare Advantage Plan and Health System Vertical Integration, 2011-2020

**DOI:** 10.1001/jamanetworkopen.2024.23733

**Published:** 2024-07-19

**Authors:** Geronimo Bejarano, Andrew Ryan, Amal Trivedi, Kendra Offiaeli, David J. Meyers

**Affiliations:** 1 Department of Health Services, Policy, and Practice, Brown University School of Public Health, Providence, Rhode Island; 2 Providence Veterans Affairs Medical Center, Providence, Rhode Island

## Abstract

This cross-sectional study compares the beneficiary and plan characteristics and trends in enrollment, premiums, star ratings, and benefits of nonintegrated, non–legacy-integrated, and legacy-integrated Medicare Advantage plans between 2011 and 2020.

## Introduction

Enrollment in Medicare Advantage (MA) has recently surpassed traditional Medicare,^1^ and MA plans have been vertically integrating with health systems.^2^ Integrated MA plans might be beneficial if aligned incentives improve quality and efficiency or detrimental if premiums increase without added value for beneficiaries.^2,3,4,5^ Legacy-integrated MA plans may differ in ways that allow them to uniquely increase quality and premiums compared with newer integrated MA plans.^5^

Due to limited data on integration status, there is a lack of evidence about the recent increase in integrated MA plans, their organizational characteristics, and the types of beneficiaries they enroll. Using a novel MA integration dataset, we compared the beneficiary and plan characteristics and trends in enrollment, premiums, star ratings, and benefits of nonintegrated, non–legacy-integrated, and legacy-integrated MA plans between 2011 and 2020.

## Methods

In this cross-sectional study, using publicly available MA data, we identified the integration status of all MA contracts using websites, marketing materials, tax data, and news reports and then validated our dataset with other datasets (eMethods in Supplement 1). Beneficiaries were considered to be in legacy-integrated MA plans if they were in Kaiser Permanente, Intermountain Health, or Geisinger plans. We linked these data to the Medicare Beneficiary Summary File (MBSF) for beneficiary characteristics and publicly reported plan data for plan characteristics. We limited our analysis to beneficiaries who were in a MA plan in January of each year. We described the beneficiary (age, race and ethnicity [RTI International race code in MBSF], sex, dual beneficiary status) and plan characteristics (premium; star rating; maximum out of pocket; hierarchical condition category [HCC] risk score; monthly capitated payments; and dental, hearing, and vision benefits) stratified by integration status, compared the growth of integrated MA plans over time, and assessed trends in premiums, star ratings, and benefits. Data were analyzed from August 2023 to May 2024 using Stata, version 18. We followed the STROBE reporting guideline, and this study was deemed exempt from informed consent by the Brown University institutional review board due to the use of deidentified data.

## Results

Our sample included all MA beneficiary-years from 2011 to 2020 (n = 159 600 378). In 2020, enrollment was 19 976 932 in nonintegrated MA plans, 2 138 365 in non–legacy-integrated plans, and 1 646 677 in legacy-integrated MA plans. Trends in number of beneficiaries enrolled overall increased in both non–legacy- and legacy-integrated MA plans (Figure). Enrollment in non–legacy-integrated MA plans increased compared with legacy-integrated MA plans. The share of enrollment of beneficiaries from racial and ethnic minority groups increased compared with White beneficiaries in both non–legacy- and legacy-integrated MA plans (Table). Beneficiaries in legacy-integrated MA plans were older than those in nonintegrated and non–legacy-integrated MA plans (mean [SD] age, 74.2 [8.8] years vs 72.6 [10.2] and 72.6 [10.4] years), less likely to be White (61.9% vs 69.6% and 67.8%), and more likely to be nondually eligible (89.8% vs 78.6% and 74.7%). Legacy-integrated MA plans had higher mean (SD) premiums ($54.89 [$18.30]) and star ratings (4.6 [0.5]) compared with nonintegrated (premium, $30.67 [$34.89]; star rating, 3.5 [0.6]) and non–legacy-integrated MA plans (premium, $45.80 [$39.00]; star rating, 3.6 [0.7]). Legacy-integrated MA plans had lower mean (SD) HCC risk scores (1.0 [0.1]) compared with nonintegrated (1.2 [0.4]) and non–legacy-integrated MA plans (1.2 [0.5]).

**Figure.**
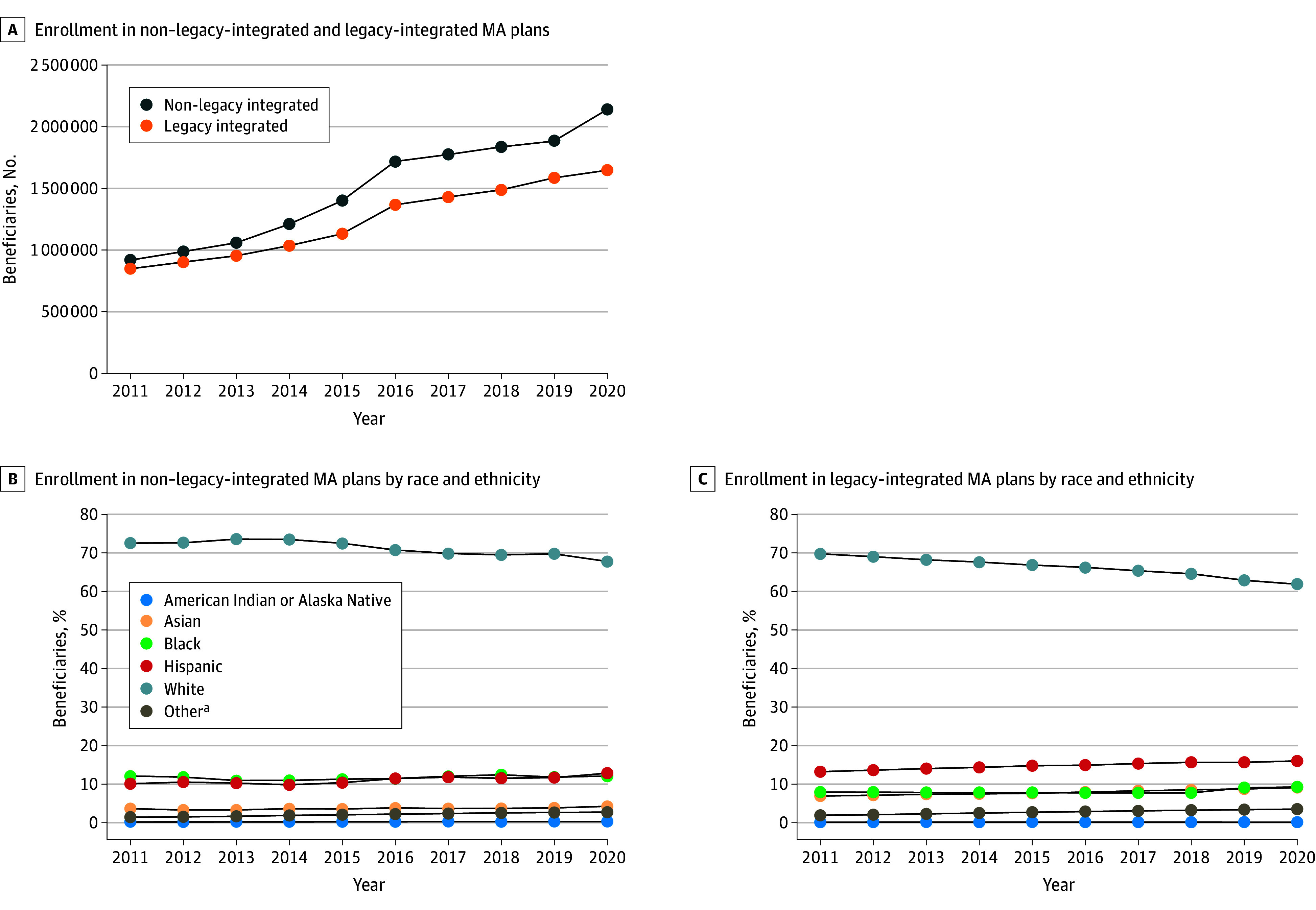
Trends in Enrollment by Integration Status and Race and Ethnicity, 2011-2020 A, Enrollment in non–legacy- and legacy-integrated Medicare Advantage (MA) plans. B, Enrollment in non–legacy-integrated MA plans by race and ethnicity. C, Enrollment in legacy-integrated MA plans by race and ethnicity. Legacy-integrated MA plans are for beneficiaries in Kaiser Permanente, Intermountain Health, or Geisinger. Non–legacy-integrated MA plans are for beneficiaries in all other integrated MA plans. The vertical integration dataset and Medicare Beneficiary Summary File datasets were used to calculate enrollment. The vertical integration dataset and Medicare Beneficiary Summary File datasets were used to calculate enrollment. The RTI International race and ethnicity variable from the Medicare Beneficiary Summary File was used for stratification. ^a^Other includes any other race or ethnicity not included in the options.

**Table.** Beneficiary and Plan Characteristics of MA Beneficiaries Stratified by Plan Integration Status in 2020

Variable	No. (%)		
	Nonintegrated MA plan	Non–legacy-integrated MA plan	Legacy-integrated MA plan
**Beneficiary characteristics**			
Enrollment			
2011	8 088 942	917 981	848 045
2020^a^	19 976 932	2 138 365	1 646 677
Age, mean (SD), y	72.6 (10.2)	72.6 (10.4)	74.2 (8.8)
Race and ethnicity			
American Indian or Alaska Native	46 364 (0.2)	5627 (0.3)	2465 (0.2)
Asian	706 534 (3.6)	90 963 (4.2)	150 290 (9.1)
Black	2 704 262 (13.5)	258 877 (12.1)	152 628 (9.3)
Hispanic	2 178 525 (10.9)	274 829 (12.9)	264 028 (16.0)
Non-Hispanic White	13 904 210 (69.6)	1 449 526 (67.8)	1 019 047 (61.9)
Other^b^	437 037 (2.2)	58 543 (2.7)	58 219 (3.5)
Sex			
Male	8 690 487 (43.5)	931 055 (43.5)	722 566 (43.9)
Female	11 286 444 (56.5)	1 207 310 (56.5)	924 111 (56.1)
Medicaid eligibility			
Full dual	2 592 669 (13.0)	404 946 (18.9)	145 294 (8.8)
Partial dual	1 674 715 (8.4)	135 618 (6.4)	22 490 (1.4)
Nondual	15 709 548 (78.6)	1 597 801 (74.7)	1 478 893 (89.8)
**Plan characteristics**			
Contracts			
2011	397	65	7
2020^a^	636	145	7
Premium			
Mean (SD), $	30.67 (34.89)	45.80 (39.00)	54.89 (18.30)
Zero premium	120 (18.9)	24 (16.6)	1 (14.3)
Star rating			
Mean (SD)	3.5 (0.6)	3.6 (0.7)	4.6 (0.5)
2-2.5 Stars	3 (0.5)	2 (1.4)	0
3-3.5 Stars	130 (20.4)	31 (21.4)	0
4-4.5 Stars	119 (18.7)	37 (25.5)	3 (42.9)
5 Stars	8 (1.3)	6 (4.1)	4 (57.1)
No rating	376 (59.1)	69 (47.6)	0
Maximum out of pocket, mean (SD), $	5286.64 (1378.23)	4865.42 (1474.24)	5500.00 (1081.66)
HCC risk score, mean (SD)	1.2 (0.4)	1.2 (0.5)	1.0 (0.1)
Per-member per-month payment, mean (SD), $	822.29 (71.28)	813.83 (110.28)	829.18 (45.44)
Dental benefits, any	345 (68.6)	95 (66.4)	3 (42.9)
Hearing benefits, any	352 (70.0)	95 (66.4)	2 (28.6)
Vision benefits, any	387 (76.9)	105 (73.4)	3 (42.9)

Abbreviations: HCC, hierarchical condition category; MA, Medicare Advantage.

^a^ Percentages were calculated using 2020 data.

^b^ Other includes any other race or ethnicity not included in the options.

## Discussion

Nearly 1 in 6 MA beneficiaries were enrolled in an integrated MA plan in 2020. Enrollment in legacy- and non–legacy-integrated MA plans has been associated with an increase in beneficiaries from racial and ethnic minority groups. Legacy-integrated MA plans had higher star ratings but higher premiums compared with nonintegrated and non–legacy-integrated MA plans. These findings are similar to previous studies.^2,3,4,6^ A limitations of this study is that it is descriptive, and causality cannot be assumed. This study suggests that only legacy- but not non–legacy-integrated MA plans offer beneficiaries higher star ratings for the higher premium compared with nonintegrated MA plans.

## Notes

Supplement 1.
eMethods.

Supplement 2.
Data Sharing Statement

## References

[1] Ochieng N, Biniek JF, Freed M, Damico A, Neuman T. Medicare Advantage in 2023: enrollment update and key trends. KFF. August 9, 2023. Accessed November 22, 2023. https://www.kff.org/medicare/issue-brief/medicare-advantage-in-2023-enrollment-update-and-key-trends/

[2] Johnson G, Lyon ZM, Frakt A. Provider-offered Medicare Advantage plans: recent growth and care quality. Health Aff (Millwood). 2017;36(3):539-547. doi:10.1377/hlthaff.2016.0722 28264957

[3] Park S, Langellier BA, Meyers DJ. Differences between integrated and non-integrated plans in Medicare Advantage. Health Serv Res. 2023;58(3):560-568. doi:10.1111/1475-6773.14101 36376095 PMC10154150

[4] Frakt AB, Pizer SD, Feldman R. Plan-provider integration, premiums, and quality in the Medicare Advantage market. Health Serv Res. 2013;48(6, pt 1):1996-2013. doi:10.1111/1475-6773.12076 23800017 PMC3876399

[5] Welch WP, Sen AP, Bindman AB. Concentration of physician services across insurers and effects on quality: early evidence from Medicare Advantage. Med Care. 2019;57(10):795-800. doi:10.1097/MLR.0000000000001193 31415344

[6] Meyers DJ, Mor V, Rahman M. Provider integrated Medicare Advantage plans are associated with differences in patterns of inpatient care. Health Aff (Millwood). 2020;39(5):843-851. doi:10.1377/hlthaff.2019.00678 32364859

