# Supplementary material for: Medicare Advantage Plan and Health System Vertical Integration, 2011-2020
Source: JAMA Netw Open. 2024 Jul 19;7(7):e2423733. doi: 10.1001/jamanetworkopen.2024.23733 (PMC11259896; doi:10.1001/jamanetworkopen.2024.23733)
Supplement: Supplement 1. — eMethods. [file jamanetwopen-e2423733-s001.pdf]

Supplementary Online Content

Bejarano G, Ryan A, Trivedi A, Offiaeli K, Meyers DJ. Medicare Advantage plan and health system vertical integration, 2011-2020. *JAMA Netw Open.* 2024;7(7):e2423733. doi:10.1001/jamanetworkopen.2024.23733

eMethods.

This supplementary material has been provided by the authors to give readers additional information about their work.

© 2024 Bejarano G et al. *JAMA Network Open.*

eMethods

Race and Ethnicity Variable

We used the race and ethnicity variable from the Medicare Beneficiary Summary File that is developed by RTI International. This variable uses race and ethnicity data from Social Security along with an algorithm based on beneficiary surname to enhance the accuracy of race and ethnicity classifications. These classifications include American Indian/Alaska Native, Asian, Black, Hispanic, White, and other. All beneficiaries not included in the five categories are included in the "other" category. We included race and ethnicity in our analysis due to the potential for enrollment differences by race and ethnicity from the social and structural determinants that may impact them.
Reference: Lines LM, Humphrey J. Imputing race/ethnicity: part 1. RTI International. September 2, 2021. Accessed May 9, 2024. https://www.rti.org/insights/imputing-raceethnicity-part-1

Integration Status:

To construct our dataset of integrated MA contracts, first we assessed every unique MA contract between 2011 and 2020 to assess if that contract was a part of an integrated system (1,233 unique contracts were evaluated). For each MA contract, we assessed vertical integration status through a combination of the contract's website and marketing materials, publicly available tax documents, and news reports. For each contract we also reported what year the integration occurred either as the year the contract was formed if integrated from inception or the first reported merger between the contract and a provider. After identifying each integrated contract, we used marketing materials and tax documents to further compile a list of all provider IDs that are included in the system (primarily CMS CCN IDs and TINs). We assigned each combination of providers and plans with a unique system ID for use in our dataset. When assessing the integration of contracts, we classified them into three categories: 1) full health system integrations (where the same business entity owns both the plan and a health system that includes a hospital), 2) full provider organization integrations (where a single financial entity owns an outpatient provider group but does not include a hospital), 3) financially aligned systems (where the same company did not own both the plan and provider, but when there was a strong financial arrangement between the two). To validate the dataset, we compared our list of integrated plans and providers against several different sources including a Becker Hospital Review list, the AHRQ Compendium of US Health Systems, and the only other published work that traces the growth of integrated MA contracts over time. We considered beneficiaries in Kaiser Permanente, Intermountain Health, and Geisinger integrated MA plans to be in legacy integrated MA plans. All others were considered to be in non-legacy integrated MA plans.
