# Supplementary material for: Medicare Advantage Plan and Health System Vertical Integration, 2011-2020
Source: JAMA Netw Open. 2024 Jul 19;7(7):e2423733. doi: 10.1001/jamanetworkopen.2024.23733 (PMC11259896; doi:10.1001/jamanetworkopen.2024.23733)
Supplement: Supplement 2. — Data Sharing Statement [file jamanetwopen-e2423733-s002.pdf]

# Data Sharing Statement

Bejarano. Medicare Advantage Plan and Health System Vertical Integration, 2011-2020. *JAMA Netw Open*. Published July 19, 2024. doi:10.1001/jamanetworkopen.2024.23733

**Data**
**Data available:** No

**Additional Information**
**Explanation for why data not available:** Data used in this study were granted under a data-use agreement with the US Centers for Medicare & Medicaid Services and cannot be provided without its consent. The dataset of vertically integrated plans is available upon request via email to the corresponding author. Code used in the calculation of these results are available from the corresponding author on request.
